# Fracture Resistance Analysis of 3D-Printed Polymers

**DOI:** 10.3390/polym12020302

**Published:** 2020-02-02

**Authors:** Ali Zolfagharian, Mohammad Reza Khosravani, Akif Kaynak

**Affiliations:** 1 School of Engineering, Deakin University, Geelong, Victoria 3216, Australia; akaynak@deakin.edu.au; 2 Chair of Solid Mechanics, University of Siegen, Paul-Bonatz-Str. 9-11, 57068 Siegen, Germany; mohammadreza.khosravani@uni-siegen.de

**Keywords:** mechanical fracture, 3D printing, mixed mode fracture, load-carrying capacity

## Abstract

Three-dimensional (3D)-printed parts are an essential subcategory of additive manufacturing with the recent proliferation of research in this area. However, 3D-printed parts fabricated by different techniques differ in terms of microstructure and material properties. Catastrophic failures often occur due to unstable crack propagations and therefore a study of fracture behavior of 3D-printed components is a vital component of engineering design. In this paper, experimental tests and numerical studies of fracture modes are presented. A series of experiments were performed on 3D-printed nylon samples made by fused deposition modeling (FDM) and multi-jet fusion (MJF) to determine the load-carrying capacity of U-notched plates fabricated by two different 3D printing techniques. The equivalent material concept (EMC) was used in conjunction with the J-integral failure criterion to investigate the failure of the notched samples. Numerical simulations indicated that when EMC was combined with the J-integral criterion the experimental results could be predicted successfully for the 3D-printed polymer samples.

## 1. Introduction

Three-dimensional printing (3D) has increasingly become one of the prevalent manufacturing methods for the development of tools and functional parts, particularly in the sectors entailing extensive customization, such as medical and aerospace [1,2,3]. However, there are currently various types of 3D printing techniques, including fused deposition modeling (FDM), stereolithography (SLA), and multi-jet fusion (MJF) that bring quite a number of uncertainties to the prediction of the outcome based on the mechanical engineering concepts [4,5].

Generally, 3D-printed parts fabricated by different techniques differ in terms of strength, stiffness, microstructure and material properties, so that it is necessary to investigate their behaviors through both experimental tests and finite element analysis (FEA) [6,7,8,9,10]. Fused deposition modeling 3D printing has shown promising capabilities to improve the specific mechanical properties of parts—such as fracture toughness—by optimization of extrusion deposition trajectories [11]. It is reported that the filament direction optimization contributed to a large deformation zone leading to “ductile-like behavior”, which subsequently caused a slow crack propagation rate [9]. Also, using 3D printing for developing co-continuous composite or interpenetrating phase composite parts proved a significant increase of damage tolerance and fracture toughness compared to conventionally manufacture composite parts [12]. Rationally designed interpenetrating architectures enabled implementing diverse techniques of crack-deflection and crack-bridging to enhance fracture resistance in 3D-printed composites. Mechanical reliability is an essential aspect of various engineering applications. Catastrophic failures often occur due to unstable crack propagations and therefore, study of fracture of components is a vital component of engineering design. In this respect, to address the fracture behavior of 3D-printed plastic components we used FDM and MJF techniques to manufacture notched samples and supported the experimental observations with a failure model.

Pre-existing cracks in different materials can be induced by various circumstances. For instance, in composite materials defects can inadvertently be produced in the service life and also during the manufacturing process [13]. In ductile materials, cracks that originate from surface imperfections or notches often grow slowly, accompanied by significant plastic deformation. However, the rate of crack growth accelerates with the progressively increasing applied stresses [14].

A reliable method for evaluating the notched ductile parts before using them in real applications, i.e., grooves, threads, and shaft holes, was developed through fracture toughness experiments [15]. In earlier studies, mode I and mixed mode conditions have been practically used to determine the fracture behavior of materials [16,17,18]. Mode I fields are tensile stress normal to the plane of the crack symmetric with respect to the crack line, while the mode II fields are shear stress acting parallel to the plane of the crack and perpendicular to the crack front. Thus, the line ahead of the crack tip has the mixed mode I/II stress intensity factors. In these studies, the finite fracture theory was employed to investigate the fracture of rounded-tip notched samples with various notch radii and inclination angles under tensile loading. However, analysis of the fracture in ductile materials are more critical due to the plastic deformations as well as the stored elastic strain energy around the notch prior to the crack initiation [19]. For the analysis of fracture in notched ductile specimens, elastic-plastic fracture mechanics is usually employed, which is complex and time consuming. Therefore, an alternative method, known as equivalent material concept (EMC), was proposed where the elastic-plastic fracture mechanics analyses are circumvented with an equivalent virtual brittle material to predict ductile failure [20,21].

In this paper, we present an analysis of the fracture of U-notched 3D-printed thermoplastic components made by two different 3D printing techniques. The equivalent material concept was employed to provide a new model in failure prediction. As the J-integral failure criterion is one of the most common brittle failure models used in the study of notched specimens, we investigated whether EMC could be combined with the J-integral failure principle to predict the fracture of U-notched 3D-printed specimens subjected to tensile loading. We present a study of the failure of U-notched 3D-printed rectangular nylon samples made by FDM and MJF under mode I and mixed mode I/II loading regimes within the framework of combined EMC and J-integral criterions. Nylon 12 filament and PA12 nylon powder were used for printing with centrally positioned notches. A series of tensile tests under static loading regime were performed, the results of which compared with that obtained by the EMC model combined with the J-integral criterion.

## 2. 3D Printing Samples

**Figure 1:**
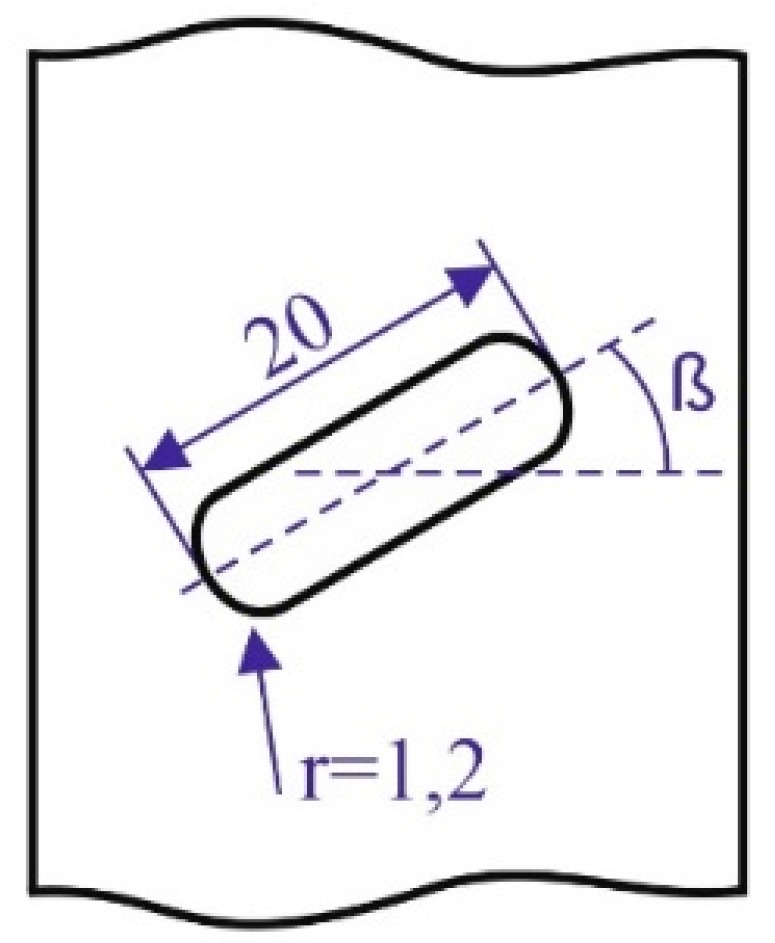
A schematic of centrally located bean-shaped notch with two U-shaped ends (dimensions in mm).

Two different types of 3D printing technologies were used to 3D print nylon samples. All the samples were first designed in a CAD platform and then saved in “.stl” format. The dogbone-type nylon samples were 3D-printed according to Type I ASTM D638 with a width of 13 mm, thickness of 5 mm and a gauge length of 50 mm as solid 100% infill rectangular plates, weakened by a centrally located bean-shaped slit with two U-shaped ends as shown in Figure 1, were 3D-printed by FDM and MJF with dimensions of 160×40×5 mm.

In FDM, nylon 12 samples were printed by a Fortus 450mc with nozzle and bed temperatures of 250 °C and 60 °C, respectively, nozzle speed 40 mm/s, infill percentage 100%, layer thickness 0.254, number of contours 1, and raster angle 0°, whereas an HP 3D printer was used to produce the MJF PA12 samples with powder melting 187 °C and infill percentage 100%. The infill percentage was 100% for both samples. The average density of samples were measured using an electronic Qualitest Densimeter SD-200L as $1.019\;\mathrm{gr}/{\mathrm{cm}}^{3}$ and $0.984\;\mathrm{gr}/{\mathrm{cm}}^{3}$ for MJF and FDM 3D-printed specimens, respectively. 

## 3. Experiments

### 3.1. Tensile Tests on 3D-Printed Dogbone Samples

**Figure 2:**
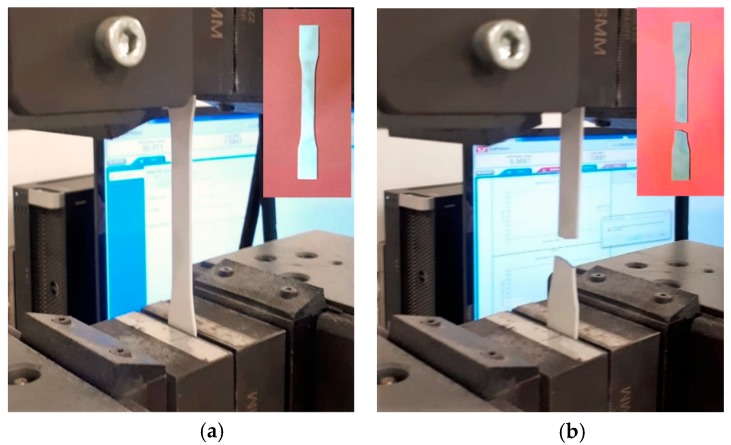
A dog-bone shaped MJF nylon 3D-printed nylon specimen; before (**a**), and after (**b**) tensile test.

**Figure 3:**
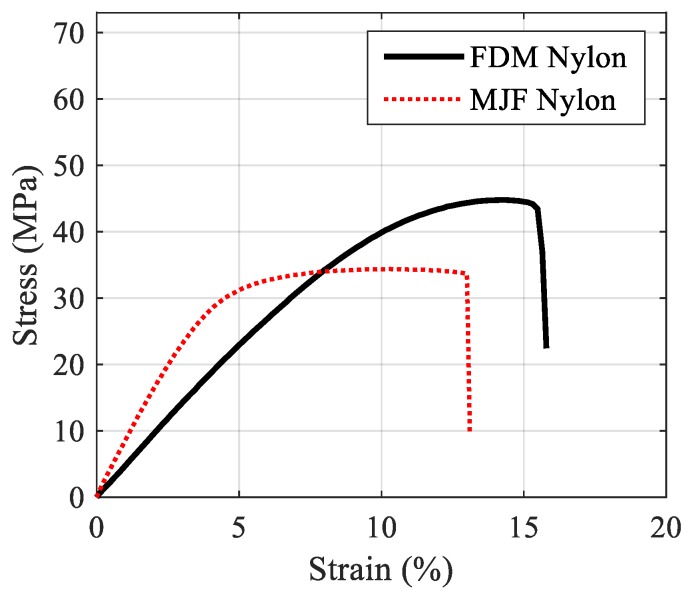
A typical tensile stress vs strain curve for the 3D-printed nylon specimens.

In order to determine basic mechanical properties, tensile tests were conducted on 3D-printed dog-bone specimens, as shown in Figure 2. The tests were performed in an Instron 300LX (Instron, High Wycombe, UK) with a crosshead speed of 5 mm/min^−1^. Tensile strength and modulus of the 3D-printed samples were determined from the stress vs strain graphs shown in Figure 3 and inserted in Table 1. For each 3D printing technique, six samples were tested to obtain the average of the mechanical properties.

The results of tensile tests showed that the average value of the modulus of elasticity of MJF 3D-printed nylon was 780 MPa, whereas the FDM sample had a lower value of 493 MPa. The average value of the percent breaking strain of the FDM and MJF samples were 16 and 13 and the average tensile strength of FDM and MJF samples were 44.8 and 34.9 MPa, respectively. Although FDM samples had lower elastic modulus, they exhibited higher tensile strength and percentage elongation compared to MJF, as well as higher modulus of toughness.

### 3.2. Fracture Tests on 3D-Printed Components

**Figure 4:**
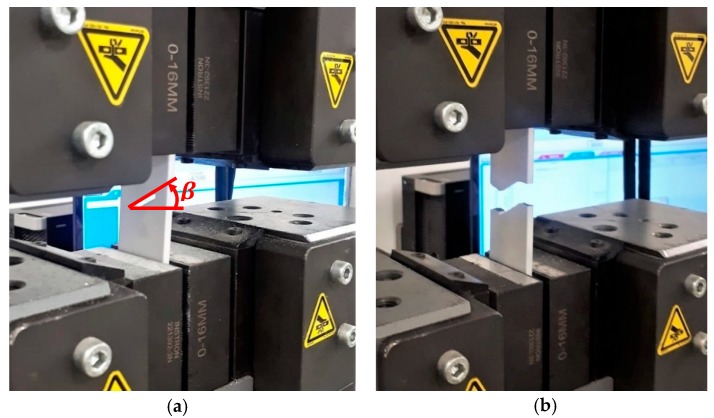
An MJF nylon 3D-printed specimen under tensile test conditions; (**a**) before (**b**) after fracture.

To investigate the behavior of samples subjected to various in-plane modes of tensile loading (i.e., mode I and mixed mode I/II loadings) as illustrated in Figure 4a,b. In mode 1 (crack opening) the axial normal stress is applied perpendicular to the plane of the notch; in mode 2 (shear mode), shear stress is applied normal to the crack front, parallel to the crack plane. In mode 3 (tearing mode), the shear stress is applied parallel to the crack front. The samples were designed and fabricated with various inclination angles (β), where β is the angle between the notch bisector line and the horizontal axis. When β is equal to zero, pure mode I loading occurs at the U-shaped ends. As the value of β increases, the in-plane shear strain develops around the U-notch which means with increasing β from zero, the loading mode on the U-notch changes from mode I towards mixed mode I/II. The oblique orientation of the notch with the axial loading gives rise to combined tensile-shear loading conditions. In the current study, we consider specific orientations of the notch at β =0°, β=30° and β=60° as well as different notch radii at r=1 mm and r=2 mm so that mode I, mixed mode I/II and notch radius effects could be investigated. The cracks are generated inside the 3D-printed samples where the load is distributed into horizontal and angled walls, to compare required fracture energy values.

**Figure 5:**
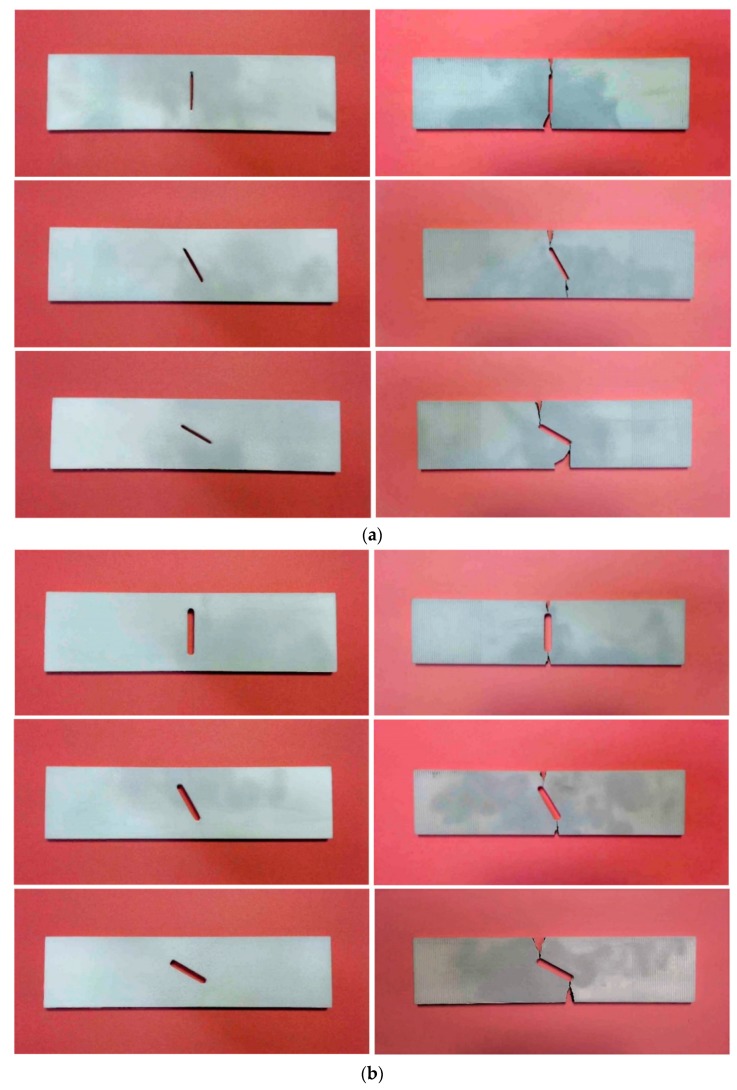

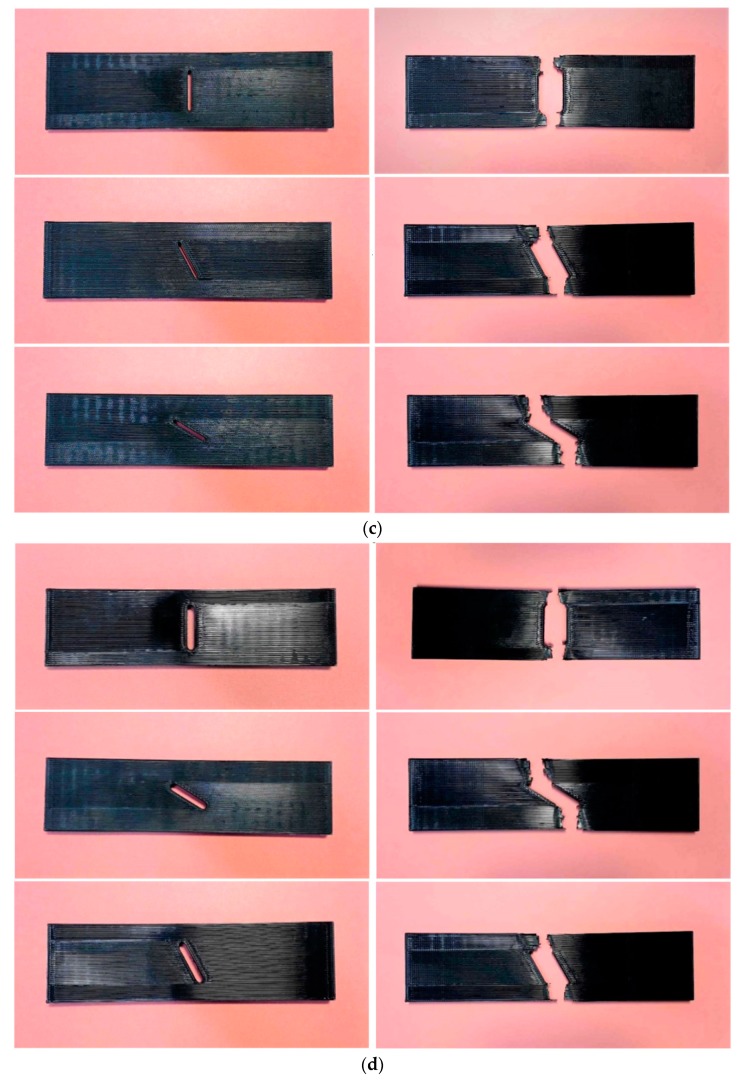
3D-printed specimens before (left) and after (right) rupture; (**a**) MJF nylon, r=1 mm, (**b**) MJF nylon, r=2 mm, (**c**) FDM nylon, r=1 mm and (**d**) FDM nylon, r=2 mm.

The 3D-printed samples prepared by aforementioned approach were axially stressed until the final failure by crack growth using an Instron 300LX (Instron, High Wycombe, UK) machine. Figure 4 shows MJF 3D-printed nylon specimen under tensile test conditions before (Figure 4a) and after fracture (Figure 4b). The tests were conducted under displacement controlled conditions at a constant cross-head speed of 20 mm/min. The photographs of the MJF- and FDM-printed nylon specimens before and after fracture at three different notch orientations and two different radii are shown in Figure 5. In each radius and inclination angle, three samples were tested to obtain the stress distribution and average of the critical load during crack growth.

**Figure 6:**
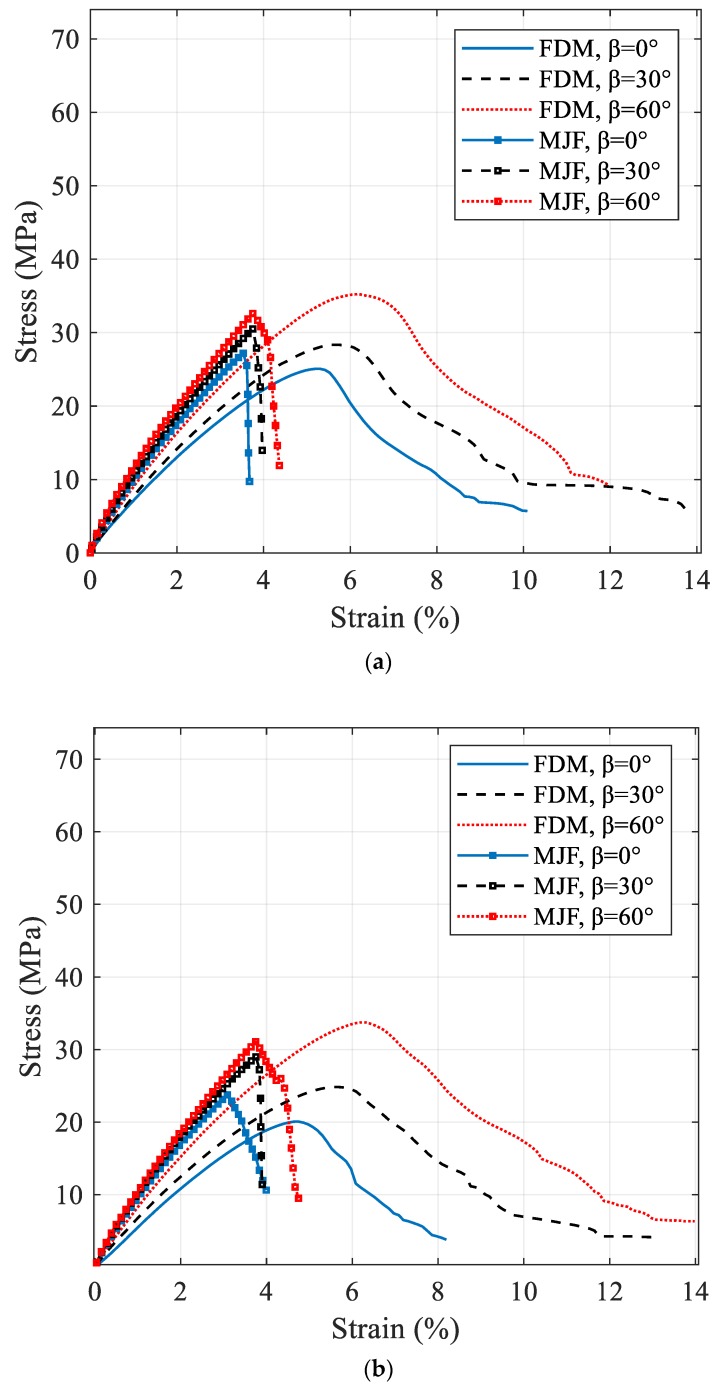
Standard tensile stress–strain curves for the FDM and MJF 3D-printed nylon; (**a**) r=1 mm, (**b**) r=2 mm

**Figure 7:**
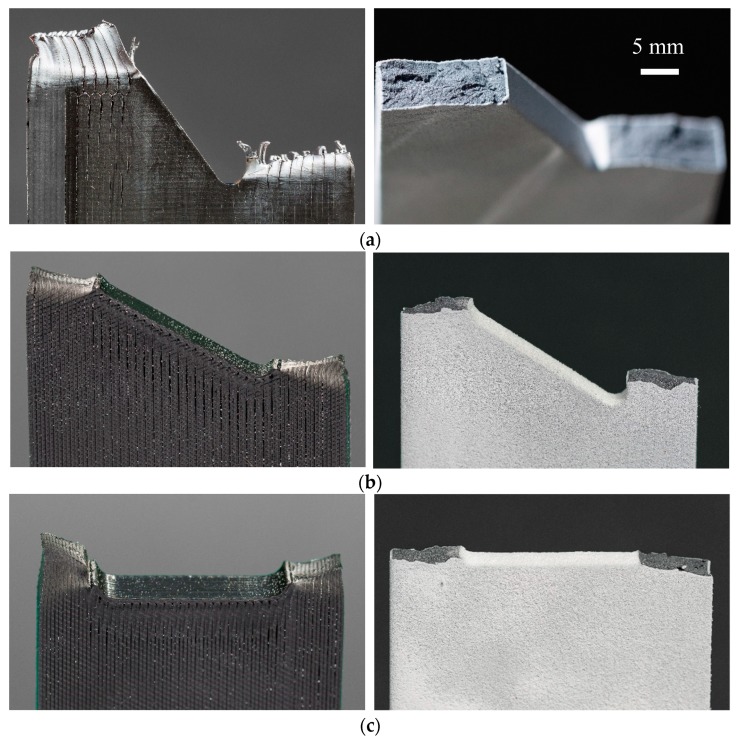
Close-up photographs FDM and MJF printed nylon specimens after fracture from left to right, respectively, with (**a**) 60°, (**b**) 30°, and (**c**) 0° notch orientations.

The representative stress–strain curves of the FDM and MJF 3D-printed nylon specimens at three different notch angles and two different notch radii are demonstrated in Figure 6. In all the cases, the ultimate tensile stress on the samples increased with an increase of inclination angle. The FDM 3D-printed nylon is clearly more ductile as it has shown approximately three times higher strain at break than MJF 3D-printed samples. The pronounced difference in tensile behavior can be attributed to the printing method employed. In the MJF method, a layer of powder is evenly distributed, then a fusing agent is applied followed by application of uniform heat to fuse the powder into an isotropic solid layer, whereas in the case of the FDM the thermoplastic filament is melted as it is positioned to form the layer. The continuous nature of the filament aligned in the direction of the applied stress resulted in better structural integrity than that achieved by fusing the polymer powder, thus resulting in significantly higher elongation at break and toughness. An examination of the close-up photographs of fractured FDM and MJF samples in Figure 7 demonstrates a ductile fracture in the case of FDM, with significant plastic deformation below the fracture surface accompanied by stress whitening. The narrowing of the cross section in the vicinity of the fracture surface indicates extensive stretching (Figure 7a,c). In contrast, close-up images of the MJF samples (Figure 7b,d) do not show any significant plastic deformation prior to failure. Irrespective of the notch orientation (Figure 5 and Figure 7), all MJF samples exhibited brittle behavior with flat fracture surfaces. Considering the tensile data in Table 2, it is evident that the failure load of MJF 3D-printed nylon at both radii is greater than FDM samples for *β* =0° and 30°. However, this is not the case when the inclination angle increased to 60°. In addition to the effect of crack angles; it was observed that increase in crack radius was associated with reduced critical load in both types of 3D-printed samples.

## 4. EMC, the J-Integral for U-Notched and THEIR Combination

Since connecting two or more parts is necessary in various specific designs, numerous engineering components contain notches with different shapes and sizes. Although existence of cracks is usually undesirable in engineering components, notches with different shapes (e.g., O, V, and U) are often unavoidable as parts of certain design requirements. However, notches have a distinct disadvantage that is stress concentrations in their vicinity that can lead to crack initiation, and ultimately failure of the component. In this section, we describe the theory of the EMC and then briefly review the J-integral in U-notched specimens, and finally explain their combination.

### 4.1. The Equivalent Material Concept (EMC)

Due to the complex and time-consuming nature of the analysis of the failure of notched ductile materials by crack propagation the equivalent material concept (EMC) was introduced [22]. This novel theory was proposed for predicting load-carrying capacity of notched components made of ductile materials. In EMC, a ductile material with elastic-plastic behavior and a valid K-based fracture toughness (K_C_ or K_IC_) is considered to be equal to a virtual brittle material with an ideal linear elastic behavior to the breaking point. In this mode, the virtual material is assumed to have the same Poisson’s ratio, Young’s modulus and K-based fracture toughness as the real ductile material, but different tensile strength. The fracture toughness can be determined either by a direct approach, where pre-notched specimens are impact tested, or an indirect method based on the examination of the notched samples [23]. In EMC a ductile material is assumed to be equal to a virtual brittle material (with linear elastic behavior), hence we can employ brittle failure criteria in linear elastic fracture mechanics in order to predict ductile failure of notched components. According to this assumption, both materials absorb the same amount of strain energy density (SED) for the crack initiation, and the tensile strength of the equivalent material calculated.

Then the tensile strength and the fracture toughness can be simultaneously utilized in brittle fracture criteria by linear elastic analysis to predict the load-carrying capacity of notched ductile parts. As the SED absorbed by the ductile material is equal to a linear elastic SED absorbed by equivalent material, the ultimate tensile strength of equivalent material would be significantly larger than of the real ductile material. In other words, equating the two energy values would result in a larger ultimate strength in the linear stress versus strain relationship. The parameters E, $\sigma_{Y},$ $\varepsilon_{Y},\varepsilon_{c}^{\;},$ $\sigma_{u}^{*}\;\mathrm{and}$ denote elastic modulus, yield stress, yield strain, strain at crack initiation and tensile strength of the equivalent material respectively. It should be noted that the final rupture would be due to the brittleness of the material.

The power-law expression for the true stress–strain relationship in the plastic zone of the ductile material is as follows:

(1) $$\sigma =K\varepsilon_{p}^{n}$$

where σ and K are the true stress and strain hardening coefficient, respectively. Also, $\varepsilon_{p}$ and n denote the true plastic strain and the strain-hardening exponent, respectively. The total strain energy density consists of elastic and plastic components and can be written as follows:

(2) $${\left(SED\right)}_{total}=\;{\left(SED\right)}_{elastic}+{\left(SED\right)}_{plastic}=\;\frac{1}{2}\;\sigma_{Y}\;\varepsilon_{Y}+\;\int_{\varepsilon_{y}}^{\varepsilon_{p}}\sigma_{p}\;d\varepsilon_{p}$$

As $\varepsilon_{Y}=\sigma_{Y}/E$, with substitution of Equation (1) into Equation (2), results:

(3) $${\left(SED\right)}_{total}=\;\frac{\sigma_{Y}^{2}}{2E}+\;\int_{\varepsilon_{Y}}^{\varepsilon_{p}}K\varepsilon_{p}^{n}\;d\varepsilon_{p}$$

By integration, Equation (3) results in

(4) $${\left(SED\right)}_{total}=\;\frac{\sigma_{Y}^{2}}{2E}+\frac{K}{n+1}\;\;\left[\left(\varepsilon_{p}^{n+1}-\varepsilon_{Y}^{n+1}\right)\right]$$

Offset yield stress $\sigma_{Y}$ is determined from a strain value of 0.2%.

(5) $${\left(SED\right)}_{total}=\;\;\frac{\sigma_{Y}^{2}}{2E}+\frac{K}{n+1}\left(\varepsilon_{p}^{n+1}-\;\varepsilon_{Y}{}^{n+1}\right)$$

where $\varepsilon_{Y}$ is the yield strain. 

The total strain energy density at any point in the plastic region can be calculated by Equation (5). By replacing $\varepsilon_{p}$ with $\varepsilon_{c}$, where $\varepsilon_{c}$ is the value of the strain at crack initiation, the total strain energy density associated with the onset of crack initiation can be determined:

(6) $${\left(SED\right)}_{total}=\;\;\frac{\sigma_{Y}^{2}}{2E}+\frac{K}{n+1}\left(\varepsilon_{c}^{n+1}-\;\varepsilon_{Y}{}^{n+1}\right)$$

Considering Equation (1), $\varepsilon_{c}$ is determined as follows:

(7) $$\varepsilon_{c}=\;{\left(\frac{\sigma_{c}}{K}\right)}^{1/n}$$

By substituting Equation (7) into Equation (6), we have

(8) $${\left(SED\right)}_{total}=\;\;\frac{\sigma_{Y}^{2}}{2E}+\frac{K}{n+1}\;\left({\left(\frac{\sigma_{c}}{k}\right)}^{n+1/n}-\;{\left(\varepsilon_{Y}\right)}^{n+1}\right)$$

In EMC, the equivalent material is considered to be a virtual brittle material with the same value of elastic modulus and plane-strain fracture, but unknown value of ultimate tensile strength. The SED for this material at the onset of crack initiation is equal to:

(9) $${\left(SED\right)}_{EMC}=\;\;\frac{\varepsilon_{u}^{**2}\;}{2E}$$

As it was mentioned earlier, in EMC, it is assumed that SED values of real ductile and the virtual brittle material are equal. Hence, Equations (8) and (9) are identical. Therefore

(10) $$\frac{\varepsilon_{u}^{**2}\;}{2E}=\frac{\sigma_{Y}^{2}}{2E}+\frac{K}{n+1}\;\left({\left(\frac{\sigma_{c}}{k}\right)}^{n+1/n}-\;{\left(\varepsilon_{Y}\right)}^{n+1}\right)$$

Finally, the tensile strength $\sigma_{u}^{*}$ of the equivalent material can be extracted as follows:

(11) $$\sigma_{u}^{*}=\sqrt{\sigma_{Y}^{2}+\;\frac{2EK}{n+1}\;\left({\left(\frac{\sigma_{c}}{k}\right)}^{n+1/n}-\;{\left(\varepsilon_{Y}\right)}^{n+1}\right)\;}$$

Material fracture toughness and the tensile strength $\sigma_{u}^{*}$ of the equivalent material are recognized as two necessary inputs in various brittle fracture criteria, to predict the crack initiation from the notch in ductile components under tensile loading regime.

### 4.2. A Brief Review of J-Integral in U-Notches

Existence of cracks in materials and their subsequent propagation under applied stresses often lead to unexpected failures, causing reliability issues in engineering structures and uncertainties over expected service life. Understanding the influence of notches and cracks of various sizes, shapes, with respect to applied stresses and material properties are important to predict the reliability of engineering components. To address this, failure criteria have been developed to predict the load-carrying capacities of engineering materials [24,25,26]. For ductile materials under general yielding condition energy dissipation [27] or J-integral criteria [28] are common powerful methods of analysis of crack propagation. In J-integral method a contour integral is defined which calculates the strain energy release rate needed to create two new surfaces in the cracked component under loading. Subsequently, some modifications of the J-integral concept were applied to different loading conditions [29,30,31]. The conditions for the path independence of the J-integral in U-and V-notched specimens [32], an analytical calculation of the J-integral for a sample with a crack initiated from a notch under elastic-plastic loading [33], and an expansion of J-integral concept considering volume element at the border of a sharp V-notch [34] were investigated.

**Figure 8:**
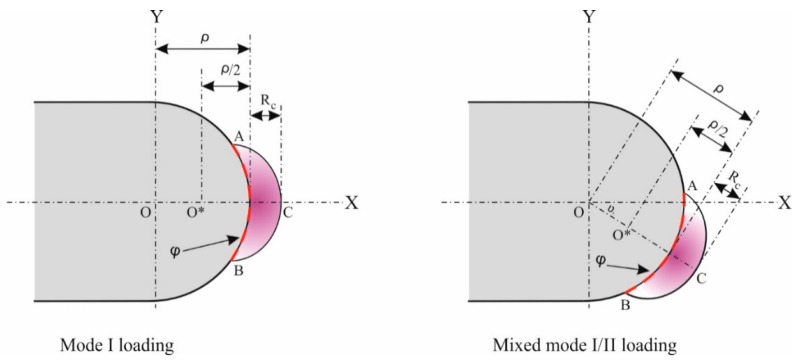
Material dependent control volume for a U-notch under mode I (**left**), and mixed mode I/II (**right**) loadings.

Here, we analyze 3D-printed U-notched rectangular specimens for ductile failure under mix mode I/II loading by means of J-integral criterion, which is described considering basic equations of this fracture parameter. As proposed [28], absolute value of the J-integral for the cracked components is as follows:

(12) $$J_{k}=\;\int_{\varphi }\;\left(Wn_{k}-\;T_{i}\frac{\partial u_{i}}{\partial x_{k}}\;ds\right),\;\;\;\left(k=1,2\right)$$

where $n_{k}$ is the unit vector normal to the specified contour path $\varphi ,\;\;u_{i}$ and $T_{i}$ denote displacement and traction vectors. W and $J_{k}$ are the strain energy density and the value of the J-integral, respectively. The value of the J-integral can be divided to two distinct values along specified axes. We assume *x* axis to be parallel to the notch bisector line, and located at the notch center of curvature (Figure 8). The values of two J-integral parameters $J_{1}$ and $J_{2}$ can be expressed along the coordinate axes *x* and *y* as follows:

(13) $$J_{1}=\;\int_{\varphi }\;\left(Wdy-\;T_{i}\frac{\partial u_{i}}{\partial x}\;ds\right)$$

(14) $$J_{2}=\;\int_{\varphi }\;\left(Wdx-\;T_{i}\frac{\partial u_{i}}{\partial y}\;ds\right)$$

In the case of mixed mode I/II loading scenario, both Equations (13) and (14) have specific non-zero values and the equivalent J-integral value $\left(J_{eq}\right)$ can be determined by:

(15) $$J_{eq}=\;\sqrt{J_{1}^{2}+J_{2}^{2}}$$

In calculation of the J-integral for un-notched specimen under mixed mode I/II loading conditions, the inner section of the indicated control volume, which incorporates the notch border must be considered. Berto et al. [35] determined the specific control volume for U-notches to have a crescent shape. This is illustrated in Figure 7, where position of the control volume for U- notch under mode I and mixed mode I/II can be seen. The critical radius of curvature of the notch $R_{c}$, which is dependent on material properties and the plane-strain condition, expressed as follows:

(16) $$R_{c}=\;\frac{\left(1+v\right)\left(5-8v\right)}{4\pi }\;{\left(\frac{K_{Ic}}{\sigma_{u}}\right)}^{2}$$

where $v,\;K_{Ic}$ and $\sigma_{u}$ are Poisson’s ratio, the plane-strain fracture toughness, and tensile strength, respectively. It should be pointed out that the thickness of the 3D-printed notched specimens are equal to 4 mm, which results in a significant plastically deformed zone around the notch at fracture. Because of the finite thickness and the resulting plastic zone, fracture toughness $\left(K_{c}\right)$ was considered and inserted in Table 1 instead of the plane-strain fracture toughness $\left(K_{Ic}\right)$. The use of $K_{c}$ is appropriate as it is not a material property and depends on the sample thickness and relevant to large-scale plastic deformation occurring in the 3D-printed notched nylon specimens.

As mentioned earlier, equation for the critical radius of curvature of the notch is based on plane strain conditions in brittle failure. In order to apply it to our case $K_{Ic}$ was substituted by $K_{c}$ to predict ductile failure of the U-notched 3D-printed plastic specimens.

As it is illustrated in Figure 8, the outer radius of the crescent-shaped control volume is equal to $\frac{\rho }{2}+\;R_{c}$. The path ACB is considered as the contour path for J-integral calculation. Due to the traction-free surface on the contour path ACB, the second term of J-integral equations are equal to zero (Equations (13) and (14)).

### 4.3. Combination of EMC and J-Integral

In this section, a new expression of the J-integral criterion is described which combines the equivalent material concept and the J-integral in order to predict load-carrying capacity of the U-notched 3D-printed specimens. J-integral criterion is extended by using the material properties obtained from EMC to analyze the fracture in ductile U-notched samples. According to the J-integral criterion, brittle failure occurs when the value of the J-integral over a contour path along the inner arc reaches the critical value of J-integral $\left(J_{cr}\right)$, which is linked to the critical strain energy density $W_{cr}$ through the inner arc ACB in Figure 8 as:

(17) $$J_{cr}≅\;W_{cr}\times \;\mathrm{arc}\;\left(ACB\right)$$

$W_{cr}$ can be evaluated according to

(18) $$W_{cr}=\;\frac{{\left(\sigma_{u}\right)}^{2}}{2E}$$

where $\sigma_{u}$ is the tensile strength. By considering the critical radius and critical strain energy density of the equivalent material J-integral criterion can be used for the prediction of the ductile fracture in U-notched specimens. By using the tensile strength $\sigma_{u}^{*}$ of the equivalent material instead of $\sigma_{u}$ in Equations (16) and (18) the critical radius of the $R_{CE}$, and the critical strain energy density $W_{CE}\;\mathrm{for}\;\mathrm{the}\;\mathrm{equivalent}$ material can be determined as follows:

(19) $$R_{CE}=\;\frac{\left(1+v\right)\left(5-8v\right)}{4\pi }\;{\left(\frac{K_{c}}{\sigma_{u}^{*}}\right)}^{2}$$

(20) $$W_{CE}=\;\frac{{\left(\sigma_{u}^{*}\right)}^{2}}{2E}$$

Substituting values obtained by Equation (20) into Equation (17):

(21) $$J_{CE}=\;\frac{{\left(\sigma_{u}^{*}\right)}^{2}}{2E}\times \;\mathrm{arc}\;\left(ACB\right)$$

where, $J_{CE}$ is the value of the J-integral for the tensile tested U-notched 3D-printed MJF and FDM nylon samples by utilizing the combined EMC/J-integral criterion. To achieve this, the strain energy density value along the specified contour path is needed, which can be evaluated by numerical simulation in finite element software.

## 5. Numerical Simulations and Results

With the aim to conform the experimental finding, we simulated the U-notched specimens that experienced tensile loads, and finite element analysis was performed. In this context, ABAQUS software was used to analyze the strain energy density distribution along the contour path defined in the vicinity of the notch. For this purpose, a finite element model was created for each U-notched specimen, utilizing eight-node biquadratic plane-strain quadrilateral element types. Considering the thickness of the specimens, they have been considered slender. In simulation, the meshes have been refined at the notch tip vicinity, because of high level of stress gradient. In numerical simulations, the tensile load was applied to the nodes that lie on the upper end of the modeled U-notched specimens. The nodes, which are lied on the lower end of the rectangular model, were constrained to be completely fixed.

**Figure 9:**
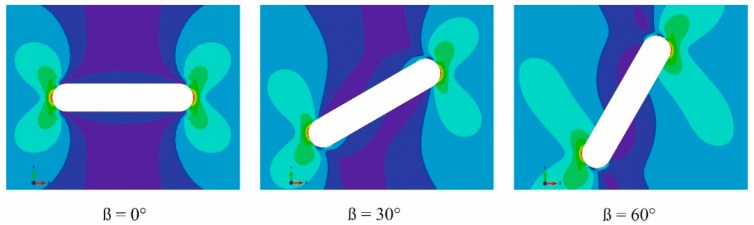
Illustration of strain energy density around the notch border for MJF nylon with a notch tip radius of 2 mm at different notch orientations.

The strain energy density contours around the notch for three selected specimens under tensile loading are illustrated in Figure 9. The parameters $J_{1}$, $J_{2}$, $J_{eq}$, arc (*ACB*), and $J_{CE}$ are presented in Table 3. It should be noted that these values are obtained for the average critical load ($P_{av}$) in the tensile experiments. It should be noted that the obtained tensile stress versus strain curve was utilized in this analysis. Considering different notch radii, there are different control volume geometries for the model. Based on the method described in the previous section, the control volume is centered along the bisector line of the notch. In addition, by considering the value of *RCE*, the value of arc (*ACB*) is obtained from FEA.

A ductile failure model, called EMC-J criterion to evaluate load-carrying capacity of a U-notched 3D-printed ductile polymer samples under mixed mode I/II loading is introduced. At first by utilizing Equation (15) the value of equivalent J-integral for an assumed load (e.g., 1 N) is determined, then the load increased till $J_{eq}$ attains $J_{CE}$. The critical load can therefore be evaluated by the following equation:

(22) $$\frac{P_{cr}}{P_{applied}}=\sqrt{\frac{J_{CE}}{\;J_{eq}}}$$

It should be pointed out that in EMC-J criterion, we considered a specified contour path round the notch border, and then theoretical results are evaluated by calculation of definite integral of SED values along this path.

**Figure 10:**
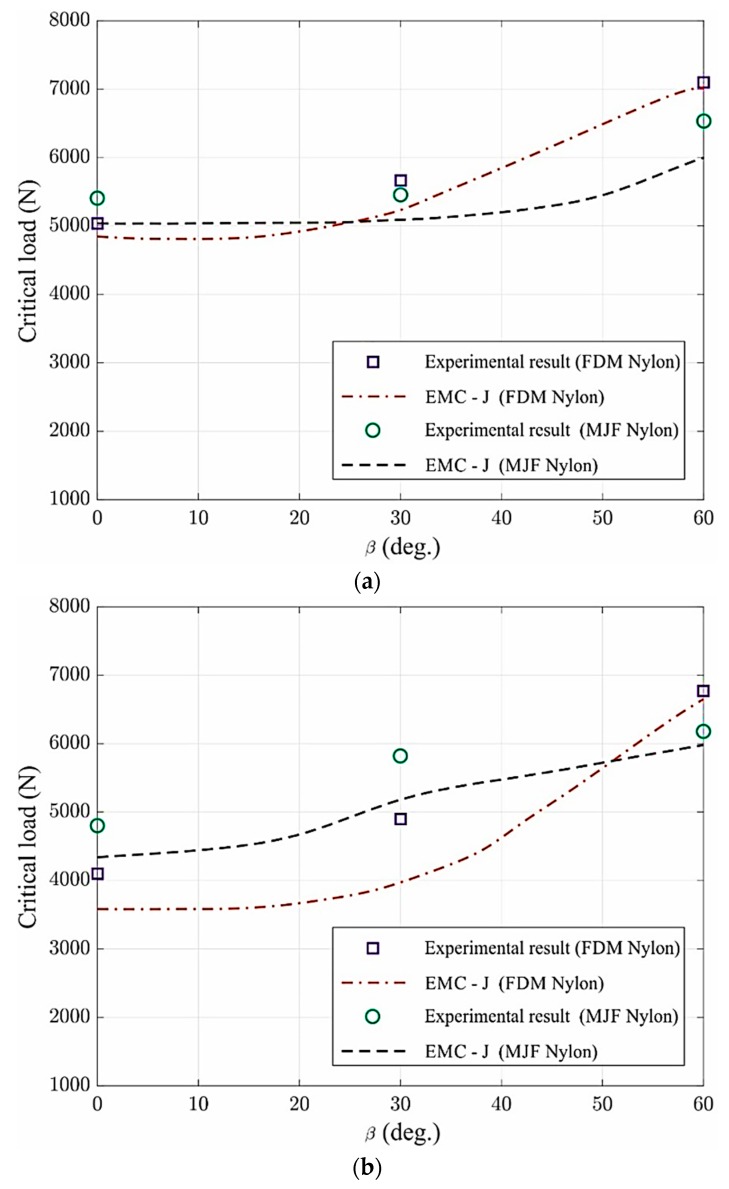
3D-printed nylon specimens U-notch comparisons via EMC-J criterion; (**a**) ρ=1 mm, (**b**) ρ=2 mm.

In the current research, EMC-J criterion is employed to predict load-carrying capacity of U-notched 3D-printed materials from a series of experiments, which were performed on notch tip radii (ρ=1 and 2 mm) with three notch inclination angles (β=0°, 30°, 60°). In Figure 10, average critical load is expressed as a function of the loading angle (β) and experimental findings are compared with results achieved by the EMC-J criterion. Results indicate that the critical load increases with the loading angle in each notch tip radius. As expected, the contribution of mode II loading increased with the increase of loading angle. That is, increase in the notch inclination angle β led to a greater plastically deformed zone around the notch at failure, manifesting as greater resistance to the applied load. As seen in Figure 10 and Table 4, the experimental findings are in good agreement with the theoretical predictions realized from EMC-J-integral criterion.

The experimental results of load-carrying capacity with theoretical predictions achieved from EMC-J criterion for the examined 3D-printed nylon specimens and differences between the experimental findings and theoretical results are presented in Table 4. There seems to a difference of up to 20%, between experimental findings and the results achieved from EMC-J, but the difference in most of the cases is lower than 8%. Therefore these findings suggest that EMC-J-integral criterion can be considered as a reliable approach to predict ductile failure of U-notched 3D-printed nylon components subjected to mixed mode I/II loading conditions.

## 6. Conclusions

There has been recent growth in the additive manufacturing of engineering components, in addition to the number of various three-dimensional (3D) printing techniques. However, the parts fabricated by different techniques differ in terms of strength, stiffness, microstructure and material properties. Therefore, investigation of fracture behaviour is a vital component of the engineering design. In this study, we presented an analysis of the fracture of 3D-printed thermoplastic components made by fused deposition modeling (FDM) and multi-jet fusion (MJF) 3D printing techniques. Nylon 12 filament and PA12 nylon powder were used for 3D printing specimens with centrally positioned notches. It was observed that the printing techniques employed resulted in pronounced differences in tensile behavior of the 3D-printed components.

In the MJF method, a uniform heat is applied to fuse the powder into an isotropic solid layer, whereas in the case of the FDM the thermoplastic filament is melted as it is positioned to form each layer. The continuous nature of the filament aligned in the direction of the applied stress have resulted in better structural integrity in the FDM samples than that achieved by MJF, thus resulting in significantly higher elongation at break and toughness. However, the mechanical properties of an FDM 3D-printed part is far more complex than it may appear at first glance. It is well established that the FDM specimens are anisotropic with the greater tensile strength in the axial direction than in the transverse direction normal to the bonds [7].

Irrespective of the notch orientations, all MJF samples exhibited brittle behavior with flat fracture surfaces. Considering the tensile tests, the failure load of MJF 3D-printed nylon was observed to be greater than the FDM samples for *β* =0° and 30°*,* while this was not the case when the inclination angle increased to 60°. In addition to the effect of crack angles, it was observed that increase in crack radius was associated with reduced critical load in both types of 3D-printed samples.

Finally, the equivalent material concept (EMC) was combined with the J-integral failure principle to predict the fracture failure of U-notched 3D-printed specimens subjected to tensile loading under mode I and mixed mode I/II loading regimes. The agreement between the experimental and simulation results proved the EMC-J approach to be capable of successfully predicting fracture in the 3D-printed notched ductile material components.

## Figures and Tables

**Table 1:** Mechanical properties of the 3D-printed nylon specimens.

Material Property	FDM Nylon	MJF Nylon
Elastic modulus, E (GPa)	0.493	0.780
Poisson’s ratio, ν	0.39	0.39
Ultimate tensile strength (MPa)	44.79	34.91
Yield stress (MPa)$,\;\sigma_{Y}$	3.61	7.21
Fracture toughness, (MPa m^0.5^)	0.92	0.58
Breaking strain (%), $\varepsilon_{B}$	16	13
Strain-hardening coefficient, *k* (MPa)	294.7138	140.9280
Strain-hardening exponent, *n* (MPa)	0.8437	0.5473
Fracture stress, $\sigma_{b}$ (MPa)	43.45	34.03

**Table 2:** The experimental failure loads of the examined 3D-printed specimens.

Material	β (deg.)	ρ (mm)	$P_{av}\;(N)$
FDM Nylon	0	1	5011.8 ± 0.4
		2	4015.1 ± 3.1
	30	1	5667.3 ± 9.5
		2	4968.6 ± 8.9
	60	1	7047.5 ± 4.7
		2	6714.7 ± 3.6
MJF Nylon	0	1	5441.2 ± 5.7
		2	4749.6 ± 2.8
	30	1	5802.8 ± 2.1
		2	5547.7 ± 1.9
	60	1	6520.2 ± 4.1
		2	6219.2 ± 3.8

**Table 3:** Numerical results of 3D-printed U-notched nylon material under mode I and mixed mode I/II loading scenarios.

Material	β (deg.)	ρ (mm)	$P_{av}$ (N)	$J_{1}$ (N/mm)	$J_{2}$ (N/mm)	$J_{eq}$ (N/mm)	arc (ACB) (mm)	$J_{CE}$ (N/mm)
FDM Nylon	0	1	5011.8	12.7	0	12.7	1.3	7.3
		2	4015.1	12.1	0	12.1	1.1	6.1
	30	1	5667.3	11.8	11.2	16.2	1.6	8.4
		2	4968.6	10.7	9.5	14.3	1.2	6.2
	60	1	7047.5	90.3	9.4	13.2	1.7	9.5
		2	6714.7	8.5	9.1	12.4	1.4	8.4
MJF Nylon	0	1	5441.2	12.9	0	12.9	1.2	5.3
		2	4749.6	12.3	0	12.3	1.1	4.8
	30	1	5547.7	11.7	11.3	16.2	1.3	5.7
		2	5802.8	10.9	9.8	14.6	1.1	4.8
	60	1	6520.2	8.2	8.7	11.9	1.4	6.2
		2	6219.2	7.4	8.1	10.9	1.2	5.3

**Table 4:** Calculated critical load for studied 3D-printed specimens by means of EMC-J criterion.

Material	β (deg.)	ρ (mm)	$P_{av}\;(N)$	$P_{EMC-J}\;(N)$	$\Delta_{EMC-J}\;(\%)$
FDM Nylon	0	1	5011.8	4726.1	5.7
		2	4015.1	3583.4	10.7
	30	1	5667.3	5218.6	7.9
		2	4968.6	3995.2	19.5
	60	1	7047.5	7003.2	0.6
		2	6714.7	6581.9	1.9
MJF Nylon	0	1	5441.2	5022.3	7.7
		2	4749.6	4362.1	8.1
	30	1	5547.7	5042.5	9.1
		2	5802.8	5245.7	9.6
	60	1	6520.2	5973.4	8.3
		2	6219.2	5912.2	4.9
